# A New Bias Error Prediction Model for High-Precision Transfer Alignment

**DOI:** 10.3390/s18103277

**Published:** 2018-09-29

**Authors:** Yutong Zhang, Shuai Yang, Shiqiao Qin, Feng Hu, Wei Wu

**Affiliations:** College of Advanced Interdisciplinary Studies, National University of Defense Technology, Deya Street 109, Changsha 410073, China; 17310040919@163.com (Y.Z.); yangshuaint@126.com (S.Y.); sqqin8@nudt.edu.cn (S.Q.); hufeng683@163.com (F.H.)

**Keywords:** inertial navigation systems (INS), transfer alignment, dynamic lever-arm, ship prediction linear motion, bias error

## Abstract

The purpose of this work was to study bias error in acceleration-based transfer alignment, which is probably caused by cross-correlation between the dynamic lever-arm and the linear motion of a ship. A new prediction model for the cross-correlation-caused error is proposed in this paper. We adopt the Bernoulli-Euler Beam as a simplified ship hull-girder model to verify the existence of the cross-correlation. Then, the mathematical mechanism and the prediction model of the bias error are deduced via the ordinary least squares theory. Three factors influence the bias error in the prediction model: the amplitude of the dynamic lever arm acceleration, the amplitude of the ship motion acceleration, and the cross-correlation between them. Simulation experiments are then conducted to test the influence of the factors. The results show that the mechanism analysis is reasonable, and the bias error prediction model is in agreement with the experimental results. Thus, the proposed prediction model has the potential to deduce the bias error in high-accuracy transfer alignment.

## 1. Introduction

Ship or an aircraft are equipped with many guided weapons equipped, such as shipboard missile defense systems and aircraft guns. In order to improve the performance of guided weapons, the method of transferring high-precision attitude information to these weapons is required. Transfer alignment (TA) is an effective approach that meets this requirement. Using accurate information from master inertial navigation systems (MINS), the orientation of the axes of the slave inertial navigation system (SINS) can be determined. 

Many research results have been already published. By comparing the outputs of the MINS with that of the SINS mounted on a guided weapon [1], the misalignment angle between SINS and MINS can be estimated using Kalman filter (KF) algorithms [2]. The TA procedures are mature and have been extensively applied in airborne, shipboard, and ground-based systems [3,4,5,6]. The acceleration matching method does not require deliberate maneuvering or integration of measurement data. Therefore, it is suitable for rapid TA in shipboard systems [7].

The lever-arm is the relative position between the two inertial navigation systems (INS) that are mounted on the same ship. Due to the lever-arm effect, the acceleration measured by the two INS conflict. This is one of the most significant challenges for acceleration-based TA, especially when the ship is maneuvering [1]. Extensive studies [8,9,10,11] have reported lever-arm modeling and compensation approaches to reduce the influence of the lever-arm effect. According to previous studies [8,10], the length of the rigid lever-arm can be measured and estimated precisely in real-time as a constant, so the lever-arm effect caused by a rigid lever-arm can be well compensated. For the dynamic lever-arm, in general, it is considered as white noise during the KF-based TA [9,11,12]. At present, most of the existing studies treated the dynamic lever-arm and the ship linear motion as two uncorrelated processes [1,13,14,15]. However, in our previous shipboard measurements and laboratory experiments, we found a significant bias error in the TA procedure when all the sensors of the INS are of high-quality and the dynamic lever-arm is considered as a second Markov process or a white noise. This suggests the existence of a method error, which is the key factor that influences the measurement accuracy of the ship deformation. Similarly, Groves et al. [16] showed that an inherent bias error exists in the attitude-based TA. Other studies [17,18,19,20] pointed out that this error is related to the correlation between ship angular motion and dynamic flexure. In order to solve this issue, Groves et al. [16] presented a mechanical model where the angular flexures is related to the specific forces and Wu et al. [21] presented a bias error model related to the phase delay between flexure and ship angular motion. However, both models cannot be directly used for acceleration-based TA. Based on these studies, we suspect that it is the correlation between the dynamic lever-arm and the ship linear motion that degrades the accuracy of TA. Currently, few studies have been published about the mechanism as well as the mathematical relationship between the correlation and the bias error in KF-based TA. Previous analyses showed that it is difficult and complex to analyze the estimation error of KF through its recursion formula [22,23], but the basic principle of the ordinary least squares (OLS) method is almost the same as that of KF and the OLS algorithm is much simpler [24,25,26]. Hence, it might be easier and more feasible to study the mechanism of bias error in KF via OLS theory.

The object of our current study was the bias error in acceleration-based TA, which is caused by the correlation between the dynamic lever-arm and the ship linear motion. In this paper, we investigate the mechanism of the bias error and present a prediction model for this error. The result of a simulation experiment shows that the bias error is related to the ratio of magnitude of the dynamic lever arm acceleration, the ship motion acceleration, and the corresponding cross-correlation coefficient.

## 2. Materials and Methods

### 2.1. TA Approach

#### 2.1.1. Introduction to the Coordinate Frame

Three coordinate frames are used in this paper, and are defined respectively as follows. The non-rotating inertial coordinate frame (*i*-frame) has origin (O_i_) at the center of the earth and is fixed with inertial space. The x,y-axes are defined in the mean equatorial plane of the earth and the x-axis points to the first point of Aries. The z-axis is directed along the mean rotation axis of the earth. The ship body coordinate frame (*b*-frame) is rigidly attached to the ship and its origin (O_b_) is at the ship’s center of gravity. The x-axis points starboard, the y-axis is in the direction of ship bow, and the z-axis points upward. The peripheral sensor body frame (*s*-frame) has origin (O_s_) at the orthogonal sensor center and the coordinates are in accordance with the sensor measurement coordinate frame.

All the frames mentioned above are established with the right hand rule.

#### 2.1.2. Acceleration Matching Function

**Figure 1:**
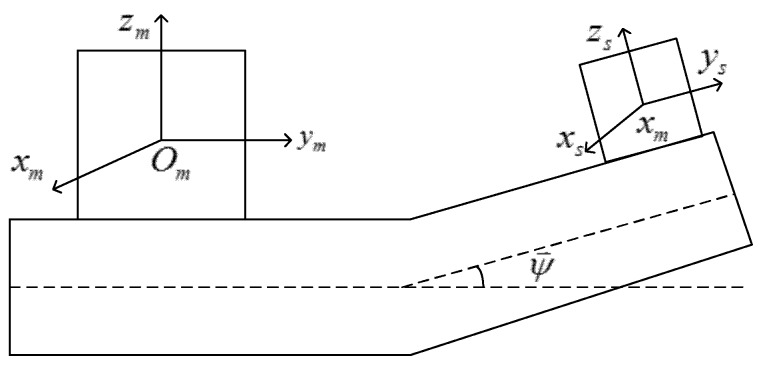
Schematic diagram of the measurement of misalignment angle.

Assuming that MINS and SINS are both equipped with three orthogonal accelerometers, as shown in Figure 1, the MINS coordinate frame O_m_(x_m_, y_m_, z_m_) is aligned with respect to the *b*-frame, and the SINS coordinate frame O_s_(x_s_, y_s_, z_s_) is in accordance with *s*-frame.

From the picture, we can see that the MINS and the SINS are equipped in different positions on the ship. Due to the ship structural dynamics, the amplitude of the misalignment Euler angle will change with time. The total misalignment Euler angle between the MINS and SINS can be defined as $\vec{\psi }$. The MINS measures the ship acceleration projected onto the O_m_(x_m_, y_m_, z_m_) coordinates, which can be written as ${\vec{f}}_{im}^{m}$, whereas the SINS measures the ship acceleration projected onto the O_s_(x_s_, y_s_, z_s_) coordinates, which can be written as. The acceleration relationship can be expressed as:

(1) $$C_{s}^{m}{\vec{f}}_{is}^{s}={\vec{f}}_{im}^{m}+{\vec{\omega }}_{im}^{m}\times {\vec{\omega }}_{im}^{m}\times {\vec{r}}_{ms}^{m}+{\dot{\vec{\omega }}}_{im}^{m}\times {\vec{r}}_{ms}^{m}+2\left({\vec{\omega }}_{im}^{m}\times {\dot{\vec{r}}}_{ms}^{m}\right)+{\ddot{\vec{r}}}_{ms}^{m}\;$$

where $C_{s}^{m}$ denotes the transform cosine matrix from the *s*-frame to the MINS’s coordinate frame with the misalignment angle, ${\vec{\omega }}_{im}^{m}$ denotes the ship inertial angular velocity projected onto the O_m_(x_m_, y_m_, z_m_) coordinates, and ${\vec{r}}_{ms}^{m}$ is the total displacement or lever-arm of SINS relative to MINS, which includes a static component ${\vec{r}}_{0}$ and a dynamic component ${\vec{r}}_{d}^{m}$. The dot operator $\dot{\left(•\right)}$ represents the derivation with respect to time *t*. If the misalignment angle $\vec{\psi }$ is small, $C_{s}^{m}$ can be written as:

(2) $$C_{s}^{m}=I+\vec{\psi }\times \;$$

where I denotes the 3 × 3 identity matrix and the operator × represents the skew symmetric cross product operator. Therefore, the difference between the accelerations measured by the MINS and SINS can be expressed as:

(3) $$\Delta \vec{f}={\vec{f}}_{is}^{s}-{\vec{f}}_{im}^{m}={\vec{f}}_{is}^{s}\times \vec{\psi }+{\vec{\omega }}_{im}^{m}\times {\vec{\omega }}_{im}^{m}\times {\vec{r}}_{ms}^{m}+{\dot{\vec{\omega }}}_{im}^{m}\times {\vec{r}}_{ms}^{m}+2\left({\vec{\omega }}_{im}^{m}\times {\dot{\vec{r}}}_{ms}^{m}\right)+{\ddot{\vec{r}}}_{ms}^{m}\;$$

where ${\vec{f}}_{is}^{s}\times$ takes the form:

(4) $${\vec{f}}_{is}^{s}\times =\left[\begin{array}{ccc} 0 & -f_{isz}^{s} & f_{isy}^{s}\\ f_{isz}^{s} & 0 & -f_{isx}^{s}\\ -f_{isy}^{s} & f_{isx}^{s} & 0 \end{array}\right]\;$$

The last four terms on the right side of Equation (3) are additional accelerations caused by the lever arm effect of the *s*-frame relative to the *b*-frame. This part should be compensated in a high-accuracy TA. For simplicity, these additional accelerations are denoted by ${\vec{f}}_{LA}$:

(5) $${\vec{f}}_{LA}={\vec{\omega }}_{im}^{m}\times {\vec{\omega }}_{im}^{m}\times {\vec{r}}_{ms}^{m}+{\dot{\vec{\omega }}}_{im}^{m}\times {\vec{r}}_{ms}^{m}+2\left({\vec{\omega }}_{im}^{m}\times {\dot{\vec{r}}}_{ms}^{m}\right)+{\ddot{\vec{r}}}_{ms}^{m}\;$$

In practice, the static component of ${\vec{r}}_{ms}^{m}$ can be measured accurately, so the lever-arm effect caused by ${\vec{r}}_{ms}^{m}$ can be well compensated. Consequently, the additional acceleration caused by the dynamic lever-arm ${\vec{r}}_{d}^{m}$ takes the form:

(6) $${\vec{f}}_{LAd}={\vec{\omega }}_{im}^{m}\times {\vec{\omega }}_{im}^{m}\times {\vec{r}}_{d}^{m}+{\dot{\vec{\omega }}}_{im}^{m}\times {\vec{r}}_{d}^{m}+2\left({\vec{\omega }}_{im}^{m}\times {\dot{\vec{r}}}_{d}^{m}\right)+{\ddot{\vec{r}}}_{d}^{m}\;$$

where ${\vec{f}}_{LAd}$ denotes the additional acceleration caused by the dynamic lever-arm, ${\dot{\vec{r}}}_{d}^{m}$ and ${\ddot{\vec{r}}}_{d}^{m}$ are the dynamic lever-arm velocity and acceleration of SINS relative to MINS, respectively. According to Petovello, M.G. [19], the frequency of ship angular motion and dynamic lever-arm ranges from 0.02 to 0.2 Hz, and the magnitude of ship’s angular velocity ${\vec{\omega }}_{im}^{m}$ ranges from 1.4 to 36.3 mrad/s. As a result, the cross product of ${\vec{\omega }}_{im}^{m}$ and ${\vec{r}}_{d}^{m}$ are far less than ${\ddot{\vec{r}}}_{d}^{m}$. In order to simplify the analysis of the influence caused by dynamic lever-arm in TA, the first three terms of Equation (6) are neglected. Equation (6) can then be approximated as:

(7) $${\vec{f}}_{LAd}\approx {\ddot{\vec{r}}}_{d}^{m}\;$$

Consequently, Equation (3) takes the form”

(8) $$\Delta \vec{f}={\vec{f}}_{is}^{s}-{\vec{f}}_{im}^{m}={\vec{f}}_{is}^{s}\times \vec{\psi }+{\ddot{\vec{r}}}_{d}^{m}\;$$

When considering the bias error of accelerometer of SINS, Equation (8) can be written as”

(9) $$\Delta \vec{f}={\vec{f}}_{is}^{s}-{\vec{f}}_{im}^{m}={\vec{f}}_{is}^{s}\times \vec{\psi }+{\ddot{\vec{r}}}_{d}^{m}+\vec{\nabla }\;$$

where $\vec{\nabla }$ is the accelerometer bias error of SINS.

Equation (9) can also be written in the form of scalar equations:

(10) $$\Delta f_{x}=-f_{isz}^{s}\cdot \psi_{y}+f_{isy}^{s}\cdot \psi_{z}+{\ddot{r}}_{dx}^{m}+\nabla_{x}\;$$

(11) $$\Delta f_{y}=f_{isz}^{s}\cdot \psi_{x}-f_{isx}^{s}\cdot \psi_{z}+{\ddot{r}}_{dy}^{m}+\nabla_{y}\;$$

(12) $$\Delta f_{z}=-f_{isy}^{s}\cdot \psi_{x}+f_{isx}^{s}\cdot \psi_{y}+{\ddot{r}}_{dz}^{m}+\nabla_{z}\;$$

#### 2.1.3. Kalman Filtering Formulation

In the TA process, the misalignment angle between SINS and MINS frames can be estimated using KF through the state equation and measurement equation. The measurement in Equation (9) is presented in a matrix form as:

(13) z=Hx+v 

where ***z*** is a measurement vector, ***H*** is a measurement matrix, ***x*** is a state vector, and ***v*** is a measurement error vector. The total misalignment Euler angle includes a static component $\vec{\varphi }$ and a dynamic component $\vec{\theta }$. The bias of the accelerometers of SINS is considered. The dynamic lever-arm velocity ${\ddot{\vec{r}}}_{d}^{m}$ is considered an unobservable disturbance. Then, the state vector is specified by:

(14) $$x={\left[\begin{array}{cccccccccccc} \varphi_{x} & \varphi_{y} & \varphi_{z} & \theta_{x} & \theta_{y} & \theta_{z} & {\dot{\theta }}_{x} & {\dot{\theta }}_{y} & {\dot{\theta }}_{z} & \nabla_{x} & \nabla_{y} & \nabla_{z} \end{array}\right]}^{T}\;$$

where ${\left[\phi_{x}\phi_{y}\phi_{z}\right]}^{T}$, ${\left[\theta_{x}\theta_{y}\theta_{z}\right]}^{T}$, and ${\left[{\dot{\theta }}_{x}{\dot{\theta }}_{y}{\dot{\theta }}_{z}\right]}^{T}$ are the three coordinate values of $\vec{\varphi }$, $\vec{\theta }$, and $\dot{\vec{\theta }}$, respectively. and ${\left[\nabla_{x}\nabla_{y}\nabla_{z}\right]}^{T}$ denotes the accelerometer measurement bias of the SINS in *s*-frame. Thus, according to Equation (9), the measurement matrix ***H*** takes the form:

(15) $$H=\left[\begin{array}{cccc} {\vec{f}}_{is}^{s}\times & {\vec{f}}_{is}^{s}\times & 0_{3\times 3} & I_{3\times 3} \end{array}\right]\;$$

where $0_{3\times 3}$ is the 3 × 3 zero matrix and $I_{3\times 3}$ is the 3 × 3 identity matrix.

In most studies [2,17], the dynamic components $\theta_{x}$, $\theta_{y}$, and $\theta_{z}$ are generally modeled by three uncorrelated second-order Markov process, which can be written as:

(16) $${\ddot{\theta }}_{j}+2\alpha_{j}{\dot{\theta }}_{j}+\omega_{0j}^{2}\theta_{j}=2\omega_{0j}^{}\sigma_{j}\sqrt{\alpha_{j}}e_{j}\left(t\right)\;j=x,y,z\;$$

where $\alpha_{i}$ is the damping factor, $\sigma_{j}$ is the standard deviation of $\theta_{j}$, and $e_{j}\left(t\right)$ is a Gaussian white noise with unit variance. The circular frequency $\omega_{0j}^{}$ is given by;

(17) $$\omega_{0j}^{}=\sqrt{\alpha_{j}^{2}+\beta_{j}^{2}}\;$$

where $\beta_{j}$ is the prevailing variation frequency of $\theta_{j}$.

The state equation for the KF is generally expressed as:

(18) $$\dot{x}=Fx+w\;$$

The state matrix ***F*** is given by:

(19) $$F=\left[\begin{array}{cccc} 0_{3\times 3} & 0_{3\times 3} & 0_{3\times 3} & 0_{3\times 3}\\ 0_{3\times 3} & 0_{3\times 3} & I_{3\times 3} & 0_{3\times 3}\\ 0_{3\times 3} & F_{32} & F_{33} & 0_{3\times 3}\\ 0_{3\times 3} & 0_{3\times 3} & 0_{3\times 3} & 0_{3\times 3} \end{array}\right]\;$$

where $F_{32}$ and $F_{33}$ are described as follows:

(20) $$\begin{array}{l} F_{32}=\left[\begin{array}{ccc} -\omega_{0x}^{2} & 0 & 0\\ 0 & -\omega_{0y}^{2} & 0\\ 0 & 0 & -\omega_{0z}^{2} \end{array}\right],\\ F_{33}=\left[\begin{array}{ccc} -2\alpha_{x} & 0 & 0\\ 0 & -2\alpha_{y} & 0\\ 0 & 0 & -2\alpha_{z} \end{array}\right] \end{array}$$

The 12 × 1 state noise vector ***w*** has the covariance matrix:

(21) $$E\left[ww^{T}\right]=diag\left\{\begin{array}{ccccccc} \underset{6}{\underset{︸}{0,\ldots ,0}} & 4\omega_{0x}^{2}\sigma_{x0}^{2}\alpha_{x} & 4\omega_{0y}^{2}\sigma_{y}^{2}\alpha_{y} & 4\omega_{0z}^{2}\sigma_{z}^{2}\alpha_{z} & 0 & 0 & 0 \end{array}\right\}\;$$

where *E*[•] is the expectation operator.

Utilizing the equations mentioned above, the misalignment angle between MINS and SINS frames can be estimated. In Equation (15), the dynamic lever-arm is undetermined in acceleration matching-based TA, and is usually treated as a measurement error. When processing TA with KF, the algorithm is converges well only when the measurement error is a white noise [27,28]. In other words, when the measurement error is colored noise, an estimation error will occur in KF. Generally, the dynamic lever arm in the acceleration-based TA is considered as a white noise [12,13]. However, the dynamic lever-arm is probably correlated with the ship linear motion in practice, which means the accuracy of acceleration-based TA may be degraded when treating the dynamic lever-arm as white noise.

### 2.2. Correlation Between Linear Motion and Dynamic Lever-Arm

#### 2.2.1. Bernoulli-Euler Beam Model

According to the hydroelasticity principle, the ship linear motion and dynamic lever-arm are all a response to the sea wave loads [29,30]. Generally, the Bernoulli-Euler beam is used for depicting a simplified ship hull model.

To verify the correlation between the linear motion acceleration and the dynamic lever-arm acceleration, we formulated them under a stochastic wave load.

The transverse vibration of a Bernoulli-Euler beam can be expressed as follows [31]:

(22) $$\frac{\partial^{2}Z(y,t)}{\partial t^{2}}+2B\frac{\partial Z(y,t)}{\partial t}+\frac{EI}{m}\frac{\partial^{4}Z(y,t)}{\partial y^{4}}=f\left(y,t\right)\;$$

where Z(y,t) denotes the linear displacement in the *z*-direction of the beam’s body frame, which is the same as the ship body frame; *B* is the damping constant; EI is the flexure rigidity of the beam; *m* is the mass per unit length of the beam; and f(y,t) is the excitation force per unit mass. It is reasonable to assume that the excitation force f(y,t) can be divided into two parts: the spatial part q(y) and the temporal part g(t).

(23) f(y,t)=q(y)g(t) 

Assuming that the beam length is *L*, then the undamped normal mode sin(nπy/L) can be used to describe the spatial behavior. Thus, the linear displacement Z(y,t) and the spatial part q(y) of f(y,t) can be expressed as:

(24) $$Z\left(y,t\right)=\sum_{n=1}^{\infty }Z_{n}(t)\sin\left(n\pi y/L\right)\;$$

(25) $$q\left(y\right)=\sum_{n=1}^{\infty }q_{n}\sin\left(n\pi y/L\right),n=1,2,3,\ldots \;$$

where the index *n* indicates the *n*th mode of the beam, $Z_{n}(t)$ is the *n*th temporal part of *Z* (*y, t*), and *q_n_* is a constant of the *n*th spatial part q(y). Substituting Equations (23)–(25) into Equation (22) yields:

(26) $${\ddot{Z}}_{n}\left(t\right)+2B{\dot{Z}}_{n}\left(t\right)+\varpi_{n}^{2}Z_{n}\left(t\right)=q_{n}g\left(t\right)\;$$

where $\varpi_{n}^{}$ is given by:

(27) $$\varpi_{n}^{2}={\left(n\pi /L\right)}^{4}EI/m\;$$

Then, the particular solution with zero state takes the form:

(28) $$Z_{n}\left(t\right)=q_{n}\int_{t_{0}}^{t}h_{n}\left(t-\tau \right)g\left(\tau \right)d\tau \;$$

where the impulse response $h_{n}\left(\tau \right)$ is given by:

(29) $$h_{n}\left(\tau \right)=\frac{e^{-Bt}\sin\left({\varpi^{\prime }}_{n}t\right)}{\varpi_{n}}\;$$

in which ${\varpi^{\prime }}_{n}=\sqrt{\varpi_{n}^{2}-B^{2}}$.

By letting *t*_0_ tend to negative infinite, Equation (28) can be rewritten as:

(30) $$Z_{n}\left(t\right)=q_{n}\int_{0}^{\infty }h_{n}\left(\tau \right)g\left(t-\tau \right)d\tau \;$$

Then, the linear displacement in the *z*-direction of the beam’s body frame Z(y,t) can be written as:

(31) $$Z\left(y,t\right)=\sum_{n=1}^{\infty }\sin\left(n\pi y/L\right)q_{n}\int_{0}^{\infty }h_{n}\left(\tau \right)g\left(t-\tau \right)d\tau \;$$

#### 2.2.2. Beam Linear Motion and Dynamic Lever-arm Model

For a specific Bernoulli-Euler beam, the normal mode sin(nπy/L) and the impulse response $h_{n}\left(\tau \right)$ are all determined according to the inherent characteristics of the beam. Thus, g(t) determines the temporal part of Z(y,t). To make the analysis more concise, without loss of generality, the temporal part *g*(*t*) takes the form:

(32) g(t)=Asin(Ωt) 

where *ω* is the circular frequency of the excitation force and *A* is the amplitude of the force.

The introduction of Equations (29) and (32) into Equation (30) yields:

(33) $$Z_{n}\left(t\right)=\frac{q_{n}A}{\varpi_{n}}\int_{0}^{\infty }e^{-Bt}\sin\left({\varpi^{\prime }}_{n}\tau \right)\sin\left(\Omega t-\Omega \tau \right)d\tau \;$$

Through which the result can be expressed as:

(34) $$Z_{n}\left(t\right)=\frac{q_{n}A}{2\varpi_{n}}C\cos\left(\Omega t+\vartheta \right)\;$$

where *C* and ϑ are all constants that only related to $\varpi_{n}$, *B*, and Ω. The two constants *C* and ϑ can be defined as:

(35) $$\begin{array}{cc} C\cos\left(\Omega t+\vartheta \right) & =\frac{\sqrt{1+a^{2}}}{B-a^{2}}\cos\left(\Omega t-\arctan\left(a\right)\right)\\ & -\frac{\sqrt{1+b^{2}}}{B-b^{2}}\cos\left(\Omega t+\arctan\left(b\right)\right) \end{array}$$

where $a={\varpi^{\prime }}_{n}-\Omega$ and $b={\varpi^{\prime }}_{n}+\Omega$. Substitute Equation (34) into Equation (24), the linear displacement in the *z*-direction of the beam’s body frame can be expressed as:

(36) $$Z\left(y,t\right)=\sum_{n=1}^{\infty }\frac{q_{n}AC}{2\varpi_{n}}\cos\left(\Omega t+\vartheta \right)\sin\left(n\pi y/L\right)\;$$

**Figure 2:**
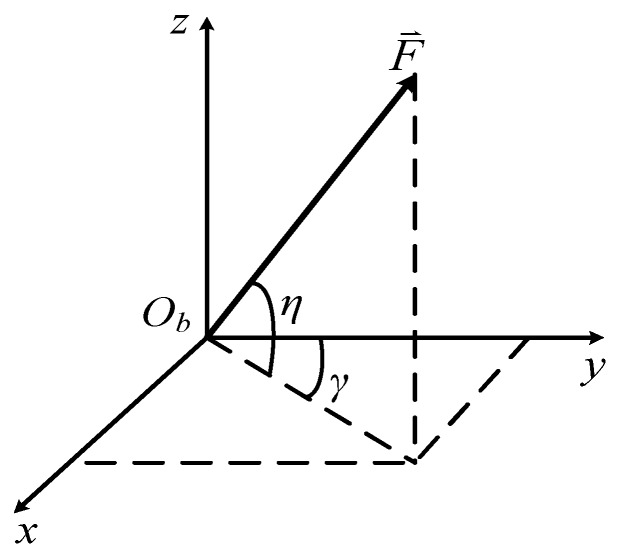
Illustration of space interaction force $\vec{F}$.

For simplicity and tractability reasons, only a one-dimensional case was derived above. Obviously, this method can be extended to three-dimensional cases. First, the space interaction force with respect to the *b*-frame is presented in Figure 2.

γ and η are used to describe the direction of $\vec{F}$, and the magnitude of $\vec{F}$ is given by:

(37) $$\left|\vec{F}\right|=q\left(y\right)\sin\left(\Omega t\right)\;$$

where q(y) and sin(Ωt) are the spatial part and temporal part of $\vec{F}$, respectively. The forces expressed in the *b*-frame take the form:

(38) $$\begin{array}{l} F_{x}=q\left(y\right)\cos\eta \sin\gamma \sin\left(\Omega t\right)\\ F_{y}=q\left(y\right)\cos\eta \cos\gamma \sin\left(\Omega t\right)\\ F_{z}=q\left(y\right)\sin\eta \sin\left(\Omega t\right) \end{array}$$

where $F_{x}$, $F_{y}$, and $F_{z}$ are the three coordinate values of $\vec{F}$. cosηsinγ, cosηcosγ, and sinη can be approximated as constants when the angle variation between $\vec{F}$ and the *b*-frame is small. According to Equation (36), the linear displacements in the *x*, *y*, and *z* directions take a similar form:

(39) $$\begin{array}{l} X_{n}\left(y,t\right)=\sum_{n=1}^{\infty }\sin\left(n\pi y/L\right)q_{n}\frac{A_{1}C_{1}}{2\varpi_{n}}\cos\left(\Omega t+\vartheta_{1}\right)\\ Y_{n}\left(y,t\right)=\sum_{n=1}^{\infty }\sin\left(n\pi y/L\right)q_{n}\frac{A_{2}C_{2}}{2\varpi_{n}}\cos\left(\Omega t+\vartheta_{2}\right)\\ Z_{n}\left(y,t\right)=\sum_{n=1}^{\infty }\sin\left(n\pi y/L\right)q_{n}\frac{A_{3}C_{3}}{2\varpi_{n}}\cos\left(\Omega t+\vartheta_{3}\right) \end{array}$$

where $A_{1}$, $A_{2}$, and $A_{3}$ represent cosηsinγ, cosηcosγ, and sinη respectively. The definitions of the other symbols are the same as the previous equations in this section.

Consequently, according to Equation (39), the acceleration of the beam’s linear motion and the relative acceleration of the linear motion between two positions *y*_1_ and *y*_2_ can be expressed as:

(40) $$\begin{array}{l} a_{x}=\ddot{X}\left(y,t\right)=-\sum_{n=1}^{\infty }\sin\left(n\pi y/L\right)q_{n}\frac{A_{1}C_{1}\Omega^{2}}{2\varpi_{n}}\cos\left(\Omega t+\vartheta_{1}\right)\\ a_{y}=\ddot{Y}\left(y,t\right)=-\sum_{n=1}^{\infty }\sin\left(n\pi y/L\right)q_{n}\frac{A_{2}C_{2}\Omega^{2}}{2\varpi_{n}}\cos\left(\Omega t+\vartheta_{2}\right)\\ a_{z}=\ddot{Z}\left(y,t\right)=-\sum_{n=1}^{\infty }\sin\left(n\pi y/L\right)q_{n}\frac{A_{3}C_{3}\Omega^{2}}{2\varpi_{n}}\cos\left(\Omega t+\vartheta_{3}\right) \end{array}$$

(41) $$\begin{array}{l} {\ddot{r}}_{x}=\ddot{X}\left(y_{2},t\right)-\ddot{X}\left(y_{1},t\right)=\sum_{n=1}^{\infty }\left[\sin\left(n\pi y_{1}/L\right)-\sin\left(n\pi y_{2}/L\right)\right]q_{n}\frac{A_{1}C_{1}\Omega^{2}}{2\varpi_{n}}\cos\left(\Omega t+\vartheta_{1}\right)\\ {\ddot{r}}_{y}=\ddot{Y}\left(y_{2},t\right)-\ddot{Y}\left(y_{1},t\right)=\sum_{n=1}^{\infty }\left[\sin\left(n\pi y_{1}/L\right)-\sin\left(n\pi y_{2}/L\right)\right]q_{n}\frac{A_{2}C_{2}\Omega^{2}}{2\varpi_{n}}\cos\left(\Omega t+\vartheta_{2}\right)\\ {\ddot{r}}_{z}=\ddot{Z}\left(y_{2},t\right)-\ddot{Z}\left(y_{1},t\right)=\sum_{n=1}^{\infty }\left[\sin\left(n\pi y_{1}/L\right)-\sin\left(n\pi y_{2}/L\right)\right]q_{n}\frac{A_{3}C_{3}\Omega^{2}}{2\varpi_{n}}\cos\left(\Omega t+\vartheta_{3}\right) \end{array}$$

where $a_{x}$, $a_{y}$, and $a_{z}$ are the three coordinates values of the beam’s acceleration, measured by the accelerometers of the INS, ${\ddot{r}}_{y}$, and ${\ddot{r}}_{z}$ denote the three coordinates values of relative acceleration between $y_{1}$ and $y_{2}$. This relative acceleration is also called as the dynamic lever-arm acceleration in Section 2. According to Equations (40) and (41), all the equations have a similar term $\cos(\Omega t+\vartheta_{j})j=1,2,3$, whereas other terms in each equation are constants for the specific beam, specific excitation force, and specific position. As a result, the correlation between the beam’s acceleration and the dynamic lever-arm acceleration is determined by $\vartheta_{j}$. When $\vartheta_{1}$, $\vartheta_{2}$, and $\vartheta_{3}$ are equal, the beam’s acceleration and the dynamic lever-arm acceleration are fully cross-correlated. Conversely, when $\vartheta_{1}$, $\vartheta_{2}$, and $\vartheta_{3}$ are not equal, the beam’s acceleration and the dynamic lever-arm acceleration are partly cross-correlated.

Notably, the Bernoulli-Euler Beam model we adopted to depict the ship null is a simplified model. Thus, we could not deduce a precise cross-correlation coefficient between the ship’s acceleration and the dynamic lever-arm acceleration basing on this model. It was difficult to obtain an accurate model owing to the complex structure of the ship. However, in this section, our main purpose was only to demonstrate the existence of the correlation between acceleration of the dynamic lever-arm and the ship’s linear motion. Therefore, the Bernoulli-Euler Beam model was sufficient. 

### 2.3. Bias Error Analysis and Modeling

#### 2.3.1. Bias Error Analysis

According to previous work [24,25,26], the basic principles of OLS and KF are almost the same, and the algorithm of OLS is much simpler than that of KF. This motivated us to study the mechanism of bias error based on OLS theory.

Equations (10)–(12) are all linear equations; therefore, the static component of the misalignment angle $\vec{\psi }$ can be easily estimated using the OLS method. Without loss of generality, it is concise and appropriate to analyze only one of the three equations as they have a symmetric form. In this section, we only consider the Equation (11) and the misalignment angle in the *x* and *z* directions estimated by the OLS method take the form [24,25,26]:

(42) $${\left[\begin{array}{cc} {\hat{\psi }}_{x} & {\hat{\psi }}_{z} \end{array}\right]}^{T}={\left(X^{T}X\right)}^{-1}X^{T}Y\;$$

where the symbol ^ means the calculated value with respect to real value, and ***X*** and ***Y*** are given by:

(43) $$X={\left[\begin{array}{cc} f_{isz1}^{s} & -f_{isx1}^{s}\\ f_{isz2}^{s} & -f_{isx2}^{s}\\ f_{isz3}^{s} & -f_{isx3}^{s}\\ \ldots & \ldots \\ f_{iszn}^{s} & -f_{isxn}^{s} \end{array}\right]}_{n\times 2},Y={\left[\begin{array}{c} Z_{y1}\\ Z_{y2}\\ Z_{y3}\\ \ldots \\ Z_{yn} \end{array}\right]}_{n\times 1}\;$$

where index *n* indicates the amount of the time series data. From Gauss’s assumption, we know that only when the random disturbances ${\ddot{r}}_{dy}^{m}$ and variable $f_{isj}^{s}(j=x,z)$ in Equation (11) are uncorrelated will the estimation of $\psi_{z}$ and $\psi_{x}$ be unbiased.

From Section 3, the dynamic lever-arm acceleration ${\ddot{\vec{r}}}_{d}^{m}$ and the acceleration ${\vec{f}}_{is}^{s}$ are likely to be cross-correlated. Supposing that the random disturbances ${\ddot{r}}_{dy}^{m}$ and variable $f_{isx}^{s}$ are correlated, at this point, ${\ddot{r}}_{dy}^{m}$ can be decomposed into two terms: one is completely correlated with $f_{isx}^{s}$, and the other is uncorrelated with $f_{isx}^{s}$. Thus, ${\ddot{r}}_{dy}^{m}$ can be expressed by:

(44) $${\ddot{r}}_{dy}^{m}=kf_{isx}^{s}+u\;$$

where *k* is a constant and *u* represents the part of ${\ddot{r}}_{dy}^{m}$ that is uncorrelated with $f_{isx}^{s}$. The constant *k* is the bias error of OLS estimation ${\hat{\psi }}_{z}$ according to the principle of the OLS method. 

As the OLS estimation of the misalignment angle in *x* and *z* take symmetric forms, the same analysis can be completed when the random disturbances ${\ddot{r}}_{dy}^{m}$ and variable $f_{isz}^{s}$ are correlated. Correspondingly, the constant *k* is the bias error of OLS estimation ${\hat{\psi }}_{x}$.

#### 2.3.2. Bias Error Prediction Model

In order to predict and deduct the bias error in TA, we attempted to deduce the mathematical expression of *k* in this section.

The cross-correlation coefficient $\rho_{rf}\left(\tau \right)$ between ${\ddot{r}}_{dy}^{m}$ and $f_{isx}^{s}$ is defined as:

(45) $$\rho_{rf}\left(\tau \right)=\frac{cor_{rf}\left(\tau \right)}{\sqrt{cor_{r}\left(0\right)cor_{f}\left(0\right)}}\;$$

where τ is the lag, and $cor_{r}\left(0\right)$ and $cor_{f}\left(0\right)$ denote the autocorrelations of ${\ddot{r}}_{dy}^{m}$ and $f_{isx}^{s}$ respectively. $cor_{rf}\left(\tau \right)$ is the cross-correlation between ${\ddot{r}}_{dy}^{m}$ and $f_{isx}^{s}$, which is given by:

(46) $$cor_{rf}\left(\tau \right)=\underset{N\to \infty }{\lim}\frac{1}{N}\sum_{t=1}^{N}{\ddot{r}}_{dy}^{m}\left(t\right)f_{isx}^{s}\left(t+\tau \right)\;$$

where *N* denotes the amount of the time series data. Substituting Equation (44) into Equation (46) yields

(47) $$cor_{rf}\left(\tau \right)=\underset{N\to \infty }{\lim}\frac{1}{N}\sum_{t=0}^{N}kf_{isx}^{s}\left(t\right)f_{isx}^{s}\left(t+\tau \right)+u\left(t\right)f_{isx}^{s}\left(t+\tau \right)\;$$

When the lag τ=0, Equation (47) is:

(48) $$cor_{rf}\left(0\right)=\underset{N\to \infty }{\lim}\frac{1}{N}\sum_{t=0}^{N}k{\left(f_{isx}^{s}\left(t\right)\right)}^{2}+u\left(t\right)f_{isx}^{s}\left(t\right)\;$$

Because *u* is uncorrelated with $f_{isx}^{s}$, Equation (48) can be simplified as:

(49) $$\begin{array}{cc} cor_{rf}\left(0\right) & =\underset{N\to \infty }{\lim}\frac{1}{N}\sum_{t=0}^{N}k{\left(f_{is}^{s}\left(t\right)\right)}^{2}\\ & =kcor_{f}\left(0\right) \end{array}$$

Finally, the model of bias error *k* can be expressed as:

(50) $$k=\rho_{rf}\left(0\right)\sqrt{\frac{cor_{r}\left(0\right)}{cor_{f}\left(0\right)}}\;$$

where $cor_{r}(0)$ represents the amplitude of ${\ddot{r}}_{dy}^{m}$, $cor_{f}(0)$ represents the amplitude of $f_{isx}^{s}$, and $\rho_{rf}(0)$ represents the relativity between these two signals. Three inferences about the bias error *k* are listed as follows: (1) The relativity between the bias error *k* and cross-correlation coefficient $\rho_{rf}(0)$ is positive. (2) The relativity between the bias error *k* and the amplitude of ${\ddot{r}}_{d}^{m}$ is positive. (3) The bias error *k* is in inverse proportion to the amplitude of $f_{is}^{s}$.

Furthermore, this new bias error prediction model has the potential to improve the accuracy of TA if the values of $cor_{r}(0)$, $cor_{f}(0)$, and $\rho_{rf}(0)$ are known.

## 3. Experiment Validation

To validate the mechanism and model of the bias error, a simulation experiment is set up in this section.

### 3.1. Simulation Conditions and Model Establishment

In the simulation, the static misalignment angle between SINS and MINS was [0.2°0.2°0.2°]*^T^*. The dynamic misalignment angles were treated as three independent second-order Markov processes whose parameters are illustrated in Equation (16). The parameters σ,β, and α used to depict the dynamic misalignment angles are listed in Table 1. The parameters were identified from the real measurement data.

According to the principle of hydroelasticity, both the linear (or angular) motion and the dynamic linear (or angular) deformation are the response of a ship to the sea wave loads [29,30]. Therefore, the ship’s inertial angular velocity, the dynamic lever-arm acceleration, and the acceleration measured by SINS can be treated as second-order Markov processes. The second-order Markov model of the ship’s inertial angular velocity, the dynamic lever-arm acceleration, and the acceleration measured by SINS can be expressed as follows:

(51) $$\begin{array}{cc} {\ddot{\omega }}_{imj}^{m}+2\alpha_{j}{\dot{\omega }}_{imj}^{m}+\omega_{0j}^{2}\omega_{imj}^{m}= & 2\omega_{0j}^{}\sigma_{j}\sqrt{\alpha_{j}}e_{j}\left(t\right)\;j=x,y,z\\ \omega_{0j}^{}= & \sqrt{\alpha_{j}^{2}+\beta_{j}^{2}} \end{array}$$

(52) $$\begin{array}{cc} {\ddot{f}}_{LAdj}+2\alpha_{j}{\dot{f}}_{LAdj}+\omega_{0j}^{2}f_{LAdj} & =2\omega_{0j}^{}\sigma_{j}\sqrt{\alpha_{j}}e_{j}\left(t\right)\;j=x,y,z\\ \omega_{0j}^{} & =\sqrt{\alpha_{j}^{2}+\beta_{j}^{2}} \end{array}$$

(53) $$\begin{array}{cc} {\ddot{f}}_{isj}^{s}+2\alpha_{j}{\dot{f}}_{isj}^{s}+\omega_{0j}^{2}f_{isj}^{s} & =2\omega_{0j}^{}\sigma_{j}\sqrt{\alpha_{j}}e_{j}\left(t\right)\;j=x,y,z\\ \omega_{0j}^{} & =\sqrt{\alpha_{j}^{2}+\beta_{j}^{2}} \end{array}$$

where all the symbols are defined in the previous section and the parameters σ,β, and α in Equations (51)–(53) are identified from our real measurement data, experimental experience, as well as the works from others [15,32]. The parameters σ, β, and α are listed in Table 2, Table 3 and Table 4, respectively. Finally, the accelerometer data of MINS were generated according to Equation 1.

The additional acceleration data generated by the static lever-arm was also generated according to Equation (5). The static lever-arm between SINS and MINS ${\vec{r}}_{0}$ was set as [5*m* 20*m* 1*m*]*^T^*. However, this additional acceleration caused by ${\vec{r}}_{0}$ was completely compensated in our simulation.

According to Equations (16), (51)–(53), every model in each direction is driven by a Gaussian white noise with unit variance. In order to investigate the bias error caused by the cross-correlation between the dynamic lever-arm acceleration ${\ddot{\vec{r}}}_{d}^{m}$ and the acceleration ${\vec{f}}_{is}^{s}$ measured by SINS, the driven noises of different models in each direction are listed in Table 5. 

With the change in the weight factor *ξ*, the correlation coefficient without lag between the driven noise of SINS acceleration data in the X- (or Z-) direction and the driven noise of the dynamic lever-arm acceleration in the Y-direction also takes a value from 0 to 1. Thus, the correlation coefficient without lag between $f_{isx}^{s}$ and ${\ddot{r}}_{dy}^{m}$ (or $f_{isz}^{s}$ and ${\ddot{r}}_{dy}^{m}$) takes a value from 0 to 1 because the second-order Markov model can be seen as a linear system.

In the simulation, all the accelerometers of MINS were assumed to be ideal. The accelerometer signals of SINS are subjected to the bias error vector ${\vec{\nabla }}_{0}$ and random walk (white noise) vector ${\vec{\nabla }}_{wn}$ Given the assumed conditions, the error parameters of the accelerometers are listed in Table 6, where SD denotes the standard deviation of the white noise.

The generated data were used to carry out the TA based on OLS or KF.

### 3.2. Result and Analysis

**Figure 3:**
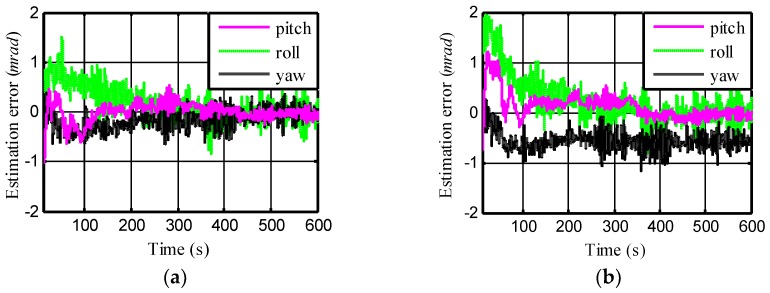
Alignment error using Kalman filtering (KF) for different correlation between the SINS acceleration data in X (or Z)-direction and the dynamic lever-arm acceleration in Y-direction: (**a**) Uncorrelated (**b**) Partly correlated.

The total data length for TA was set to 600 s with a sampling frequency of 20 Hz. First, fix all the parameters presented from Table 1, Table 2, Table 3, Table 4, Table 5 and Table 6 except for the weight factor *ξ* in Table 5. Then, as the value of *ξ* changes, a number of simulations were performed to investigate the alignment performance under different relevancies between $f_{isx}^{s}$ (or $f_{isz}^{s}$) and ${\ddot{r}}_{dy}^{m}$. The KF-based alignment results of when $f_{isx}^{s}$(or $f_{isz}^{s}$) and ${\ddot{r}}_{dy}^{m}$ were uncorrelated, i.e., *ξ* = 0, are shown in Figure 3a. 

The average alignment error caused by the dynamic lever-arm was almost zero at the end of 100 s in KF-based TA. When $f_{isx}^{s}$ (or $f_{isz}^{s}$) and ${\ddot{r}}_{dy}^{m}$ were partly correlated, i.e., *ξ* = 0.5, the average alignment error caused by the dynamic lever-arm in the yawing angle was −0.6 mrad, as shown in Figure 3b. These results show a preliminary conclusion that the bias error is related to the cross-correlation coefficient between the SINS acceleration data and dynamic lever-arm acceleration. However, it is interesting that the error of the roll was still very small, as can be seen in Figure 3b, which shows that the cross-correlation coefficient (or *ξ*) is not the only factor that determines the bias error.

In order to ensure the reliability and stability of the simulation results, all the following results were obtained by averaging over 100 independent trials in the presence of randomly generated noise signals. Similarly, the bias error of KF-based TA in each trial was calculated by averaging the estimation errors. The OLS-based TA errors are also provided to verify the reasonability of explaining the bias error mechanism through OLS theory. Furthermore, the bias error *k* was calculated to validate the correctness of the bias error prediction model shown in Equation (50).

**Figure 4:**
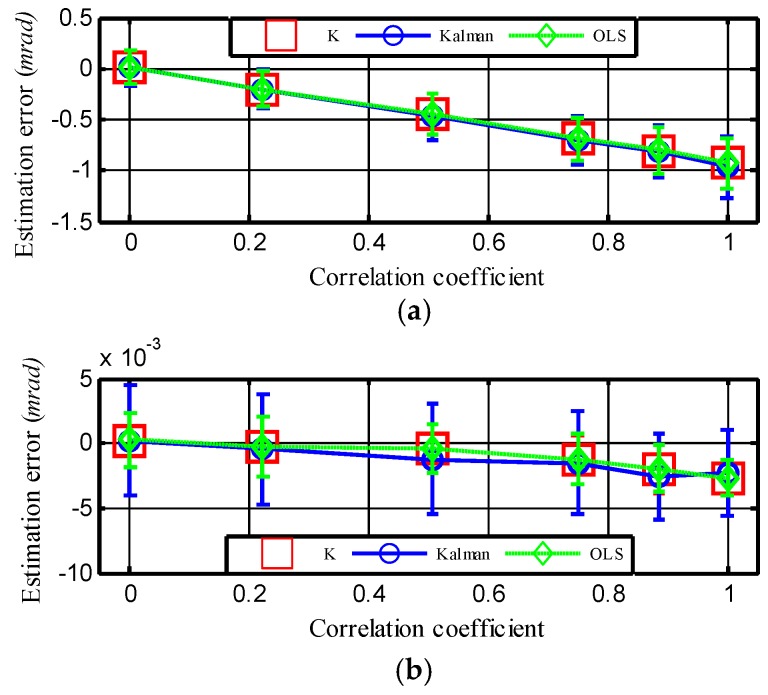
Bias error as a function of the correlation coefficient. (**a**) Error for the estimation of yawing angle. (**b**) Error for the estimation of pitching angle. The vertical lines indicate the corresponding standard deviations or error bars.

To further the investigation of the cross correlation effect on the alignment accuracy, the weight factor *ξ* was increased from 0 to 1 with a step length of 0.2. The average bias errors caused by the dynamic lever-arm are shown in Figure 4, where the vertical lines indicate the related standard deviations. It can be seen from Figure 4a that the bias error increased from −0.01 mrad to −0.9 mrad as the correlation coefficient between $f_{isx}^{s}$ and ${\ddot{r}}_{dy}^{m}$ increased from 0 to 1. This result coincides with the first inference about Equation (50). However, Figure 4b shows that the bias error in the pitching angle was almost unaffected as the cross-correlation coefficient changed. A reason for this finding is that the gravity in $f_{isz}^{s}$ increased the autocorrelation of $f_{isz}^{s}$ more than that of ${\ddot{r}}_{dy}^{m}$, so the cross-correlation coefficient had little influence on the bias error in the pitch direction according to Equation (50). This result also provides an indirect proof that the bias error is related to the amplitude of ${\vec{f}}_{is}^{s}$ as the analysis in Section 4. Both the bias error caused by the dynamic lever-arm in KF-based TA and OLS-based TA were in accordance with the bias error *k*, as shown in Figure 4. This result indicates that the basic principles of OLS and KF are almost the same. Therefore, our analysis of the mechanism of the bias error and the mathematic expression of bias error *k* are reasonable.

**Figure 5:**
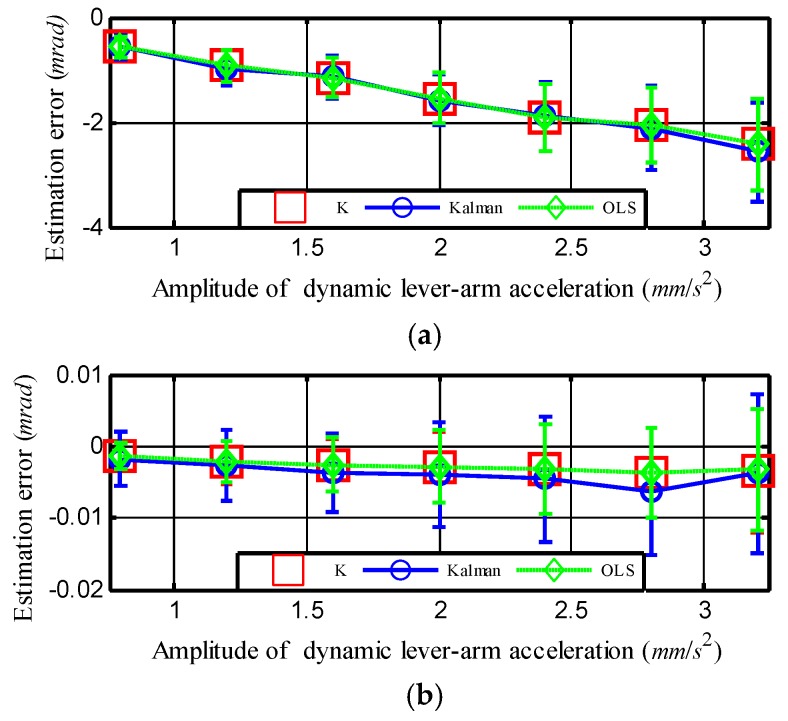
Bias error as a function of the amplitude of dynamic lever-arm acceleration ${\ddot{r}}_{dy}^{m}$. Error for the estimation of (**a**) yawing angle and (**b**) pitching angle. The vertical lines indicate the corresponding standard deviations or error bars.

Next, set *ξ* = 0.5 and fix other experimental conditions except the amplitude of ${\ddot{\vec{r}}}_{d}^{m}$. The influence of the amplitude of ${\ddot{\vec{r}}}_{d}^{m}$ on the alignment accuracy was then investigated. As presented in Equation (50), the amplitude of ${\ddot{\vec{r}}}_{d}^{m}$ is n proportional to the square root of $cor_{r}\left(0\right)$. Figure 5 depicts the corresponding bias error caused by the dynamic lever-arm as the amplitude of ${\ddot{r}}_{d}^{m}$ changes. It shows that as the amplitude of ${\ddot{r}}_{dy}^{m}$ increased from 0.8 to 3.2 mm/s^2^, the bias error in yawing angle increased from −0.6 to −2.4 mrad. This result fits the second inference about Equation (50) well. Figure 5b shows that the bias error in pitching angle changed slowly as the amplitude of ${\ddot{r}}_{dy}^{m}$ increased. Similarly, the reason for this finding is that the gravity in $f_{isz}^{s}$ makes the autocorrelation of $f_{isz}^{s}$ much higher than that of ${\ddot{r}}_{dy}^{m}$, which was discussed through Figure 4b.

**Figure 6:**
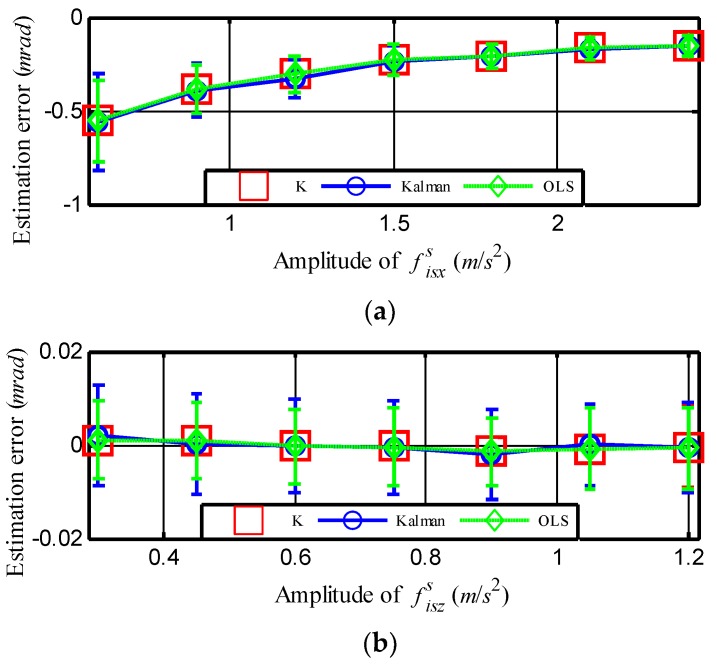
Bias error as a function of the amplitude of acceleration measured by SINS. Error for the estimation of (**a**) yawing angle and (**b**) pitching angle. The vertical lines indicate the corresponding standard deviations or error bars.

Finally, a simulation was carried out to investigate the influence on the alignment accuracy caused by the amplitude of ${\vec{f}}_{is}^{s}$. In this simulation, we set *ξ* = 0.5 and fixed other experimental conditions except for the amplitude of ${\vec{f}}_{is}^{s}$. Figure 6 depicts the corresponding bias error caused by the dynamic lever-arm as the amplitude of $f_{isx}^{s}$. $f_{isz}^{s}$ increased simultaneously by one to four times. Figure 6a shows that as the amplitude of $f_{isx}^{s}$ grew from 0.6 to 2.4 m/s^2^, the bias error in yawing angle decreased from −0.6 to −0.2 mrad. This result matches the third inference for Equation (50) well. 

Furthermore, it can be inferred from Figure 6a that the bias error in yawing angle can be reduced when the ship is maneuvering, such as accelerating or turning. However, as illustrated in Figure 6b, it was hard to obtain the bias error in lower pitching angles, even though the amplitude of $f_{isz}^{s}$ was increasing. The reason for this is that the gravity in $f_{isz}^{s}$ makes the bias error in the pitching angle already very low, which can be deduced from Equation (50).

In summary, bias error can be observed in standard TA, especially in the estimation of yawing angle, when there is a correlation between the SINS acceleration data and dynamic lever-arm acceleration. The simulation results showed that the bias error in TA is related to three factors: the amplitude of ${\ddot{r}}_{dy}^{m}$, the amplitude of $f_{isx}^{s}$, and the relationship between the two signals. The bias error is exacerbated when that coefficient or the amplitude of the dynamic lever-arm acceleration increases, whereas the accuracy of TA can be improved by increasing the linear acceleration of the ship, which means that the amplitude of SINS acceleration $f_{isx}^{s}$ increases. This conclusion agrees with Equation (50) in Section 4, suggesting that the prediction model of the bias error is reasonable. Furthermore, the coupling effects in the KF-based and OLS-based TA were almost the same, which demonstrates the reasonability of the process to analyze the mechanism of bias error in KF-based TA through the OLS theory.

## 4. Conclusions

By analyzing the cross-correlation between the dynamic lever-arm and the linear motion of a ship, a new bias error prediction model for acceleration-based TA was proposed in this paper. First, the basic theory of the TA approach was introduced. Then, the Bernoulli-Euler beam model was used to verify the existence of cross-correlation between the dynamic lever-arm acceleration and the ship liner motion acceleration. A bias error prediction model was deduced and the simulation results showed good agreement with the model. Hence, this prediction model provides a promising method to deduce bias error. For example, if the autocorrelation of the dynamic lever arm acceleration and the acceleration of the ship motion, as well as their corresponding cross-correlation coefficient can all be estimated, on the condition that the hydroelastic model, statistical analysis, as well as the finite element are fully analyzed [20], then the bias error can be calculated and compensated. In future research, we plan to carry out a feasible solution based on this work in order to further enhance the accuracy of TA.

## Figures and Tables

**Table 1:** Model parameters of dynamic misalignment angle.

	σ (mrad)	β (Hz)	α (1/s)
X-axis	0.0776	0.16	0.1
Y-axis	0.1551	0.19	0.1
Z-axis	0.1551	0.18	0.1

**Table 2:** Model parameters of ship angular velocity.

	σ (mrad/s)	β (Hz)	α (1/s)
X-axis	4.7	0.17	0.1
Y-axis	7.2	0.16	0.1
Z-axis	2.2	0.18	0.1

**Table 3:** Model parameters of dynamic lever-arm acceleration.

	σ (mm/s^2^)	β (Hz)	α (1/s)
X-axis	3.1	0.17	0.1
Y-axis	0.77	0.16	0.1
Z-axis	1.5	0.17	0.1

**Table 4:** Model parameters of acceleration measured by the accelerometers of SINS.

	σ (mm/s^2^)	β (Hz)	α (1/s)
X-axis	0.6	0.16	0.1
Y-axis	0.4	0.17	0.1
Z-axis	0.3	0.17	0.1

**Table 5:** Driven noise of different models in each direction. (WNx, WNy, and WNz denote independent Gaussian white noises in three directions, and the weight factor *ξ* takes a value from 0 to 1).

	X-Direction	Y-Direction	Z-Direction
Ship inertial angular velocity (${\vec{\omega }}_{im}^{m}$)	WNx	WNy	WNz
Dynamic misalignment angle ($\vec{\theta }$)	WNx	WNy	WNz
Dynamic lever-arm acceleration (${\ddot{\vec{r}}}_{d}^{m}$)	WNx	WNy	WNz
SINS acceleration data (${\vec{f}}_{is}^{s}$)	(1 − *ξ*)*WNx + ξ*WNy	WNy	(1 − *ξ*)*WNz + ξ*WNy

**Table 6:** Parameters of the accelerometer noises used in the simulation system.

MINS			SINS	
	Bias	White Noise	Bias (mGal)	white Noise (SD) (mGal)
X	0	0	30	10
Y	0	0	30	10
Z	0	0	30	10
